# Pleural Fluid Serum Bilirubin Ratio for Differentiating Exudative and Transudative Effusions

**Published:** 2018-06-30

**Authors:** Pawan Agrawal, Tirtha Man Shrestha, Pratap Narayan Prasad, Ramesh Prasad Aacharya, Priyanka Gupta

**Affiliations:** 1 Department of Medical Services, Bayalpata Hospital, Achham, Nepal; 2 Department of General Practice and Emergency Medicine, Maharajgunj Medical Campus, Tribhuvan University, Nepal; 3 Department of ENT-Head and Neck Surgery, Bluecross Hospital, Kathmandu, Nepal

**Keywords:** *bilirubin ratio*, *exudates*, *light's criteria*, *pleural effusion*, *transudates*

## Abstract

**Introduction:**

In pleural effusion, differentiating exudative and transudative fluid is an important clinical evaluation. The objective of the study was to determine the efficacy of pleural fluid serum bilirubin ratio in differentiating exudative and transudative effusions. In resource-limited settings with no facilities to measure lactate dehydrogenase levels, using pleural fluid bilirubin ratio may help in better clinical decision.

**Methods:**

It was a **cross sectional** study, conducted in the emergency department of Tribhuvan University Teaching Hospital. All the patients attending for emergency care with pleural effusion from 6th Jan 2015 to 5th Jan 2016 were included. The cases were divided as exudates and transudates on basis of final diagnosis. Serum and pleural fluid specimen were collected and sent for investigations. The data for various laboratory parameters especially those of lights criteria and bilirubin ratio were then analyzed and fluid nature was compared with results from parameters and final diagnoses.

**Results:**

Among 103 cases, 74 (71.84%) had exudate and 29 (28.16%) had transudate. The commonest cause of effusion was pneumonia 37 (35.92%), second being tubercular 24 (23.30%) followed by malignant effusion 13 (12.60%), congestive heart failure 12 (11.65%), chronic kidney disease 11 (10.67%) and liver cirrhosis 6 (5.82%). The mean bilirubin ratio for exudates exceeded that for transudates. Considering the cutoff point of 0.6, the sensitivity, specificity, positive predictive value and negative predictive value were respectively 88.00%, 93.00%, 97.00% & 75.00%.

**Conclusions:**

Pleural fluid serum bilirubin ratio can be utilized as a diagnostic tool for differentiating exudative and transudative effusions.

## INTRODUCTION

Pleural effusion is a common diagnostic problem encountered in patients with respiratory symptoms.^1^ Light's criteria has been accepted as a gold standard to distinguish exudate from transudate.^2^

Nevertheless, various workers over the last two decades have noted that even this criteria misclassify a significant proportion of effusion and showed that none of the parameters could be used as a sole criteria to diagnose the type of effusion with precision.^3,4^ Light's criteria misidentified 20% of transudate as exudate.^2^

The laboratories in district hospitals do not have facility for measuring lactate dehydrogenase (LDH) levels and the district physicians fail to utilize the criteria. Thus, any new parameter that guides the district physicians towards the nature of pleural effusion will prove very helpful in quality healthcare to the rural communities.

The objective of the study was to determine the efficacy of pleural fluid serum bilirubin ratio in differentiating exudative and transudative effusions and compare it with Light's criteria.

## METHODS

The study was a cross sectional study, conducted in emergency of Tribhuvan University Teaching Hospital (TUTH), Kathmandu over one year duration from August 2014 to August 2015. Ethical approval was obtained from institutional review board of institute of medicine, Tribhuvan University. The study did not involve any additional intervention to the patient besides diagnostic thoracocentesis. The study had non-probability convenience sampling technique and enrolled 121 patients after initial screening. All the patients coming to the emergency ward with chest pain, shortness of breath or generalized swelling underwent clinical and radiological screening for pleural effusion. The blood & pleural fluid specimen were collected after written informed consent for determining the study variables as well as other parameters necessary to confirm the diagnoses. The cases were followed in the inpatient departments (IPDs) to ensure their final diagnoses for effusion in case the diagnoses were not confirmed in the emergency and at home on telephone, if discharged from the emergency before their diagnoses were reached.

Since adult age groups were only taken care in the emergency department and pediatric cases were referred to other emergency ward within the hospital complex, the study had participants greater than fifteen years of age. Only the effusions with a single diagnosis were included in the study. The final diagnoses should be able to differentiate for exudative and transudative nature of effusion. The diagnoses included were pneumonia, tuberculosis, congestive heart failure, chronic kidney disease and liver cirrhosis. The cases with two concomitant diagnoses like pneumonia and congestive heart failure were excluded because each of them can produce different type of effusion. Similarly, the diagnosis like hypothyroidism which could cause borderline effusion were avoided.^5^ Thus, 103 cases fulfilled the inclusion criteria. Among 18 excluded cases, 15 had more than one diagnoses concomitantly and three were of occult malignancy. All the data collected were entered and assimilated using SPSS statistical analysis software. The sensitivity, specificity, positive predictive value and negative predictive value of pleural fluid serum bilirubin ratio and other lab parameters in determining the exudates were calculated by Bayesian method. The differences between the two groups in bilirubin ratio and others were analyzed using Student's ‘t’ test.

## RESULTS

**Figure 1.**
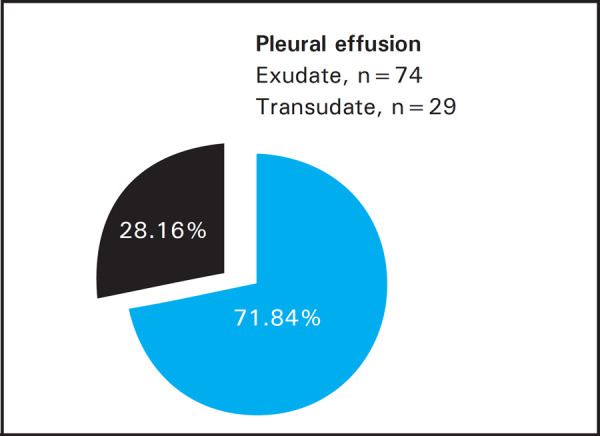
Distribution of types of pleural effusion.

In this study of 103 cases, 74 (72%) cases were exudates and 29 (28%) were transudates based upon their final diagnoses (Figure 1).

**Figure 2.**
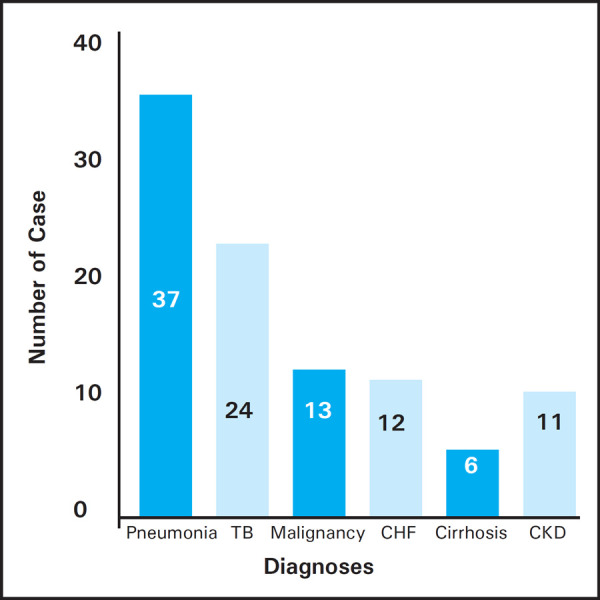
Causes of pleural effusion.

The commonest cause of pleural effusion was pneumonia 37 (35.92%) followed by tuberculosis 24 (23.30%), lung malignancies 13 (12.62%), congestive heart failure 12 (11.65%), chronic kidney disease 11 (10.68%) and liver cirrhosis 6 (5.82%) (Figure 2).

**Figure 3.**
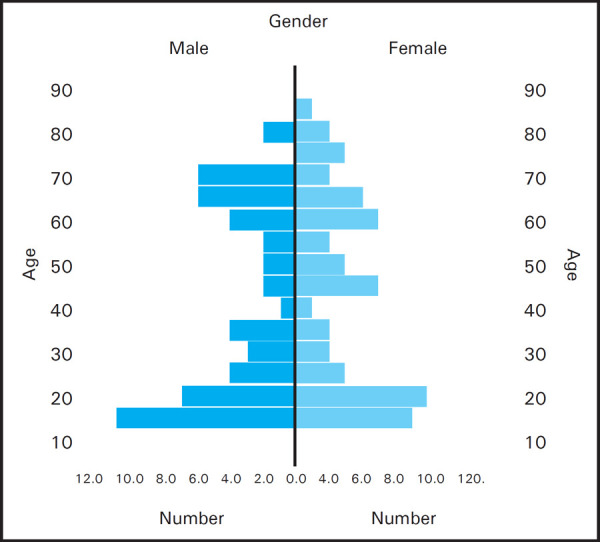
Age gender distribution.

The study had 52 (50.49%) males and 51 (49.51%) females. The prevalence was higher among young adults as illustrated (Figure 3).

The sensitivity, specificity, positive predictive value and negative predictive value of various parameters were analysed using Bayesian method, the gold standard being the known diagnoses (Table 1). The sensitivity, specificity, positive predictive value and negative predictive value of bilirubin ration were 88%, 93%, 97% and 75% respectively.

**Table 1.** Sensitivity, specificity, positive predictive value (PPV) and negative predictive value (NPV) of laboratory parameters.

S.N.	Parameters	Sensitivity	Specificity	PPV	NPV
1.	Pleural fluid to serum bilirubin ratio	88.00%	93.00%	97.00%	75.00%
2.	Pleural fluid serum protein ratio	90.59%	89.65%	95.89%	86.66%
3.	Pleural fluid to serum LDH ratio	89.18%	89.65%	95.65%	76.47%

No single parameter differentiated the type of effusion with infallible accuracy. Table 2 showed the number of exudative and transudative effusions misplaced in the other category when the parameter was used.

**Table 2.** Table depicting number of cases misclassified by each parameter.

S.N.	Parameters	Exudates n (%)	Transudates n (%)
1.	Pleural fluid serum protein ratio	4 (5.40%)	3 (10.34%)
2.	Pleural fluid to serum LDH ratio	8 (10.81%)	3 (10.34%)
3.	Pleural fluid to serum bilirubin ratio	9 (12.16%)	2 (6.89%)

## DISCUSSION

Pleural effusion needs to be analyzed with respect to its nature to work out the diagnosis. And this study aimed to facilitate this process in resource limited settings. The study possessed 74 exudates (71.84%) and 29 transudates (28.16%). The observed prevalence of exudates was in accordance with other studies so far.^2,4,6^ Nevertheless Meisel et al had equal number of exudates & transudates in their study.^7^ The commonest cause of effusion was pneumonia 35.92%, second being tubercular 23.30% followed by lung malignancy 12.60%, congestive heart failure 11.65%, chronic kidney disease 10.67% and liver cirrhosis 5.82%. These data suggested the majority of effusions were mainly due to lung infections. Light et al. had 28.66% of malignant effusion being the top most, 26.00% of congestive heart failure, 17.33% parapneumonic effusions, 9.33% tubercular, 3.33% cirrhosis and 2% chronic kidney disease.^2^ The increased number of malignant cases and cardiac failure and decreased number of infective cases were obvious in scenario of western population. Hamal et al concluded tubercular to be the foremost cause of effusion 33.90%, second being the malignant one 14.50% followed by parapneumonic effusion 11.30%.^6^ The observed difference was explained partly by the fact that the study of Hamal et al was conducted in inpatients unlike this study. The cases of TB & malignancy were gradual in developing effusion so that patients got habituated and present to the OPDs and got admitted for evaluation unlike in pneumonia where the fever and acute shortness of breath with sudden effusion compelled the patients to rush into the emergency department. The mean age in exudates was 39 and in transudates was 52. The difference was statically significant. This implies that likelihood of transudative effusions increases with advancing age which can be explained by the fact that the heart failure, cirrhosis and chronic kidney disease often affect the late age groups. Among 103 cases of pleural effusions, 52 were males and 51 were females. The male female ratio was 1.05 in exudates group and 0.93 in the transudates group. The study did not support the predominance of male population in having pleural effusion. The finding contrast with that of other studies. The paper of Prabhakaran R et al had 58% males and 42% females.^7^ Likewise, Rao obtained 44 males and 11 females in his study.^8^ Sunanda et al also revealed that the male patients out numbered females in cases of pleural effusion.^9^

Since protein ratio was being used widely, researchers had been interested in assessing the efficiency of pleural fluid serum bilirubin ratio. The efficacy was comparable to that of pleural fluid protein and pleural fluid serum protein ratio. The mean bilirubin ratio for exudates exceeded that for transudates. The observed difference was statistically significant, P value from t test being <0.05. Considering the cutoff point of 0.6, the sensitivity, specificity, positive predictive value and negative predictive value were respectively 88%, 93%, 97% & 75%. Meisel et al revealed the sensitivity of 96% and specificity of 83%.^10^ Nine of the exudates (12.16%) and two of the transudates (6.89%) were misclassified using bilirubin ratio as the differentiating criteria. With light's criteria, eight of the exudates (10.81%) failed to meet the criteria and were labelled as transudates whereas three of the transudates (10.34%) fulfilled the criteria and misplaced as exudates. In the rural setting the facility for LDH level is not easily available, the facility for bilirubin level is frequently found and can be utilized in work up for pleural effusion.

## CONCLUSIONS

The infective effusions from pneumonia and tuberculosis are more common as compared to other etiologies in emergency department. Pleural fluid serum bilirubin ratio can be useful for differentiating the nature of pleural effusion and is comparable to light's criteria in resource limited settings of rural district hospitals.
